# Structural Insights into Cellulolytic and Chitinolytic Enzymes Revealing Crucial Residues of Insect β-N-acetyl-D-hexosaminidase

**DOI:** 10.1371/journal.pone.0052225

**Published:** 2012-12-27

**Authors:** Tian Liu, Yong Zhou, Lei Chen, Wei Chen, Lin Liu, Xu Shen, Wenqing Zhang, Jianzhen Zhang, Qing Yang

**Affiliations:** 1 School of Life Science and Biotechnology, Dalian University of Technology, Dalian, China; 2 School of Software Technology, Dalian University of Technology, Dalian, China; 3 State Key Laboratory of Drug Research, Shanghai Institute of Materia Medica, Chinese Academy of Sciences, Shanghai, China; 4 State Key Laboratory of Biocontrol, School of Life Sciences, Sun Yat-sen University, Guangzhou, China; 5 Research Institute of Applied Biology, Shanxi University, Taiyuan, China; Natural Resources Canada, Canada

## Abstract

The chemical similarity of cellulose and chitin supports the idea that their corresponding hydrolytic enzymes would bind β-1,4-linked glucose residues in a similar manner. A structural and mutational analysis was performed for the plant cellulolytic enzyme BGlu1 from *Oryza sativa* and the insect chitinolytic enzyme OfHex1 from *Ostrinia furnacalis*. Although BGlu1 shows little amino-acid sequence or topological similarity with OfHex1, three residues (Trp^490^, Glu^328^, Val^327^ in OfHex1, and Trp^358^, Tyr^131^ and Ile^179^ in BGlu1) were identified as being conserved in the +1 sugar binding site. OfHex1 Glu^328^ together with Trp^490^ was confirmed to be necessary for substrate binding. The mutant E328A exhibited a 8-fold increment in *K*_m_ for (GlcNAc)_2_ and a 42-fold increment in *K*_i_ for TMG-chitotriomycin. A crystal structure of E328A in complex with TMG-chitotriomycin was resolved at 2.5 Å, revealing the obvious conformational changes of the catalytic residues (Glu^368^ and Asp^367^) and the absence of the hydrogen bond between E328A and the C3-OH of the +1 sugar. V327G exhibited the same activity as the wild-type, but acquired the ability to efficiently hydrolyse β-1,2-linked GlcNAc in contrast to the wild-type. Thus, Glu^328^ and Val^327^ were identified as important for substrate-binding and as glycosidic-bond determinants. A structure-based sequence alignment confirmed the spatial conservation of these three residues in most plant cellulolytic, insect and bacterial chitinolytic enzymes.

## Introduction

Cellulose and chitin, both β-1,4-linked linear saccharides composed of glucose (Glc) or N-acetylglucosamine (GlcNAc), respectively, are the two most abundant biomasses distributed in the plant and animal kingdoms, respectively [1], [2]. The biodegradation of these saccharides proceeds via the same path, endo-enzymes first degrade higher degree polymerized saccharides into oligosaccharides and then exo-enzymes degrade oligosaccharides into monosaccharides. Cellulase (EC 3.2.1.4) and chitinase (EC 3.2.1.14) are the endo-splitting enzymes, and β-glucosidase (EC 3.2.1.21) and β-N-acetyl-D-hexosaminidase (EC 3.2.1.52) are the exo-splitting enzymes required for cellulose and chitin degradation, respectively [1], [2]. The similarity between these two biomolecules hints at a convergent evolution between the degradation enzymes from plants and chitin-containing animals. The recent crystal structural information of exo-splitting enzymes provides evidence of this linkage.

Rice (*Oryza sativa*) β-glucosidase BGlu1 (Os3BGlu7), which exhibits high activity toward cellooligosaccharides [3], belongs to glycosyl hydrolase family 1 according to the CAZy database [4]. The catalysis proceeds via a double displacement mechanism by which two acidic residues act as nucleophile and acid/base catalyst, respectively [5]. The crystal structure of BGlu1 revealed that BGlu1 possesses a classic (β/α)_8_-barrel catalytic domain constituting a substrate binding pocket with subsites for binding both the leaving Glc (-1 subsite) and the other cellooligosaccharide residues (+1 subsite, +2 subsite and so on) [6], [7]. The amino acid residues constituting the subsites for binding the cellooligosaccharide residues, in particular the +1 subsite, are thus determinants for both substrate affinity and specificity. Three residues, Trp^358^, Ile^179^ and Tyr^131^, are found to be crucial for the +1 Glc binding. Trp^358^ stacks with the +1 Glc through its indolyl group, Ile^179^ forms a hydrophobic interaction with the +1 Glc through its isopropyl group, and Tyr^131^ forms a hydrogen bond with the C3-OH of the +1 Glc through its phenolic hydroxyl group [6], [7]. In this way, the +1 and -1 sugars are stabilized and exist around a 90° dihedral angle adjacent to each other, yielding a conformation where the glycosidic bond between them becomes more susceptible to attack by the catalytic residues.

**Figure 1:**
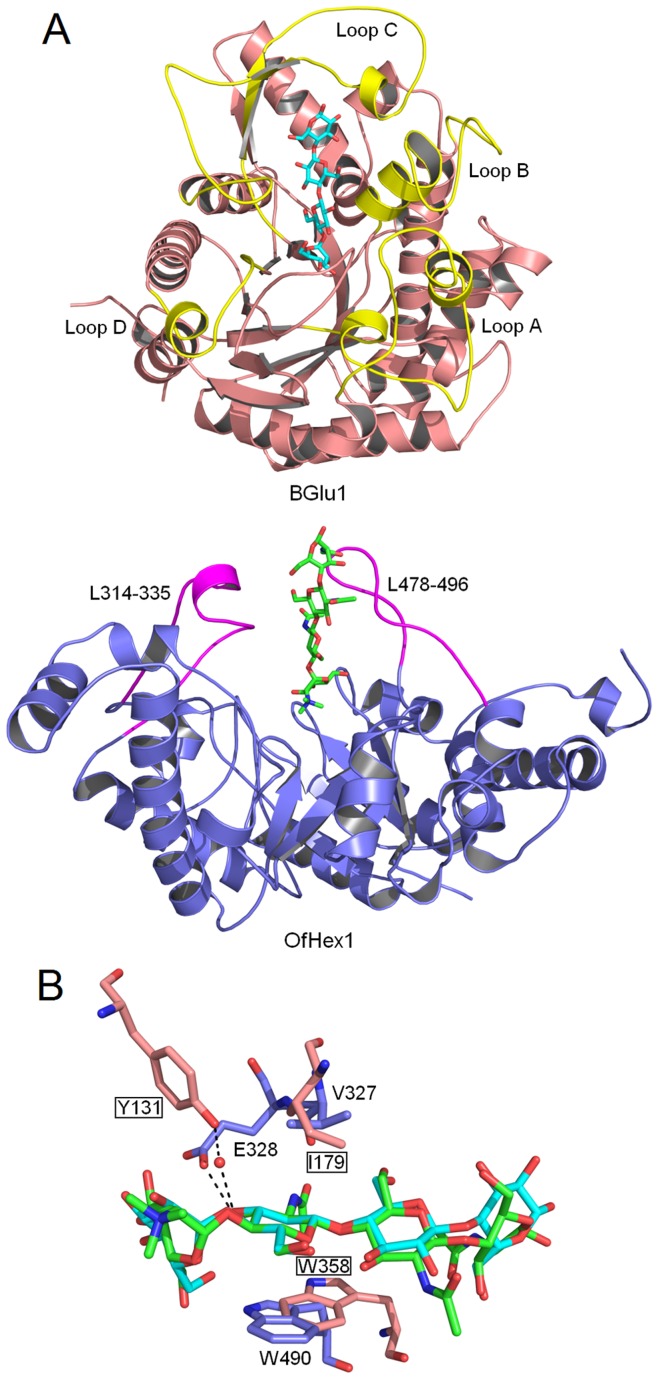
Structure comparison of BGlu1 and OfHex1. (**A**) Overall structure comparison of the catalytic domains of BGlu1 and OfHex1. The structures of the BGlu1-(Glc)_4_ complex (BGlu1 in wheat; Loop A, Loop B, Loop C and Loop D in yellow; (Glc)_4_ in cyan) and OfHex1-TMG-chitotriomycin complex (OfHex1 in blue; *L*_314–335_ and *L*_478–496_ in pink; TMG-chitotriomycin in green) were used. (**B**) Overlapping of +1 subsites in the active pockets of BGlu1 (in wheaten) in complex with (Glc)_4_ (in cyan) and OfHex1 (in blue) in complex with TMG-chitotriomycin (in green). Water molecules are shown as red balls and hydrogen bonds are shown as black dashes. The residues of BGlu1 were grounded.

The structural alignment reveals that the active pocket architecture of OfHex1 and BGlu1 is very similar although their overall topology as well as amino acid sequence shares very little similarity (Fig. 1) [8]. Insect OfHex1 from the Asian corn borer (*Ostrinia furnacalis*) is a β-N-acetyl-D-hexosaminidase specialized for chitin degradation during molting and metamorphosis [9]. This enzyme attracts much attention because of its potential as a species-specific target for developing eco-friendly pesticides [10]–[15]. OfHex1 shows high activity toward β-1,4-linked chitooligosaccharides but is not able to hydrolyse β-1,2-linked GlcNAc from *N*-glycans, thus exhibiting very high substrate specificity [9]. According to the CAZy database [4], β-N-acetyl-D-hexosaminidase belongs to family 20, and uses a substrate-assisted double displacement mechanism by which the 2-acetamido group in the substrate GlcNAc, instead of the acidic residue in a β-glucosidase, acts as the nucleophile [16], [17]. OfHex1 also contains a -1 subsite and +1 subsite for sugar moiety binding, and the three corresponding amino acid residues found in BGlu1 for binding the +1 GlcNAc are found in OfHex1(Val^327^, Glu^328^ and Trp^490^).

We thus hypothesized that both cellulolytic and chitinolytic enzymes may possess a similar mechanism to increase their affinities and specificities toward physiological substrates. In this study, a comparative structural investigation between BGlu1 and OfHex1 was performed. Although the two enzymes exhibit no similarity in either their overall structure or amino acid composition of their active pockets, crucial conserved residues were discovered by structure-based sequence alignment among cellulolytic and chitinolytic enzymes from different species. The significance of these residues was investigated by site-directed mutagenesis, biochemical characterization and crystal structure analysis. This research may provide a basis for the development of pesticides that are suitable for use in cases where plant protection is a priority.

## Materials and Methods

### Structure Comparison and Multiple Sequence Alignment

Structure comparison was performed by PyMOL (Schrödinger LLC, Portland, OR) and structural figures were also prepared by PyMOL. Multiple sequence alignments were performed using PROMALS3D [18] and the alignment figure was prepared with ESPript [19].

### Preparation of Enzymes

Mutations of OfHex1 (V327G, E328Q and E328A) were made by In-Fusion™ Advantage PCR Cloning Kit (TaKaRa) using the following primers: V327G (forward primer, 5′-GGTGAGCCCCCATGCGGTCAGCTC-3′; reverse primer, 5′-CGCATGGGGGCTCACCGCAGTATGATTTCC-3′); E328Q (forward primer: 5′- CAGCCCCCATGCGGTCAGCTCA -3′, reverse primer: 5′- ACCGCATGGGGGCTGCACGCAG -3′); E328A (forward primer: 5′-GCGCCCCCATGCGGTCAGCTCA-3′, reverse primer: 5′-ACCGCATGGGGGCGCCACGCAG-3′); W490A (forward primer: 5′-GCTTGTTCTCCTTACATCGGATGGCAG-3′, reverse primer: 5′-GATGTAAGGAGAACAAGCGTTGTTACCAGC-3′). Full-length PCR products were cloned into the expression vector pPIC9 (Invitrogen). Then the expression vector plasmids were linearized by *Pme*I (New England Biolabs) and transformed into *Pichia Pastoris* GS115 cells by electroporation. After growing on a RDB plate at 30°C for 48 h, the positive clones were selected by PCR [20].

The positive clones were cultured in BMMY broth at 30°C for 144 h, and methanol (1% of the total volume) was added every 24 h. Wild-type and mutant OfHex1 were purified from the culture supernatant by ammonium sulfate precipitation (65% saturation), affinity chromatography on a HisTrap™ HP column (5 ml, GE Healthcare) followed by anion exchange chromatography on a Mono Q™ 5/50 GL column (1 ml, GE Healthcare) [20]. The purity of the mutants was analyzed by SDS-PAGE.

The β-N-acetyl-D-hexosaminidase from *Streptomyces plicatus* (SpHex) was purchased from New England Biolabs. The β-N-acetyl-D-hexosaminidase from *Serratia marcescens* (SmChb) was expressed and purified according to the reported method [21].

### Enzymatic Assay

The enzymatic properties of wild-type and mutant forms of OfHex1, SmChb and SpHex were determined using GlcNAcβ1,4GlcNAc [(GlcNAc)_2_, Sigma] and GlcNAcβ1,2Man (Dextra Laboratories).

For the substrate (GlcNAc)_2_, the reaction mixtures contained 0.04, 0.08, 0.12, 0.16 and 0.2 mM of substrate and an appropriate amount of enzyme in 50 µl of Britton-Robinson’s wide range buffer [pH 7.0 for OfHex1, mutants of OfHex1(V327G, E328A, E328Q and W490A) and SmChb, pH 4.0 for SpHex]. For GlcNAcβ1,2Man, the reaction mixtures contained 0.03, 0.05, 0.1, 0.2 and 0.4 mM of substrate and an appropriate amount of enzyme in 50 µl of Britton-Robinson’s wide range buffer at the same pHs mentioned above. After incubation at 25°C for a specific period, 10 µl of the reaction was immediately analysed using a TSKgel amide-80 column (4.6×250 mm, Tosoh) at 25°C. The reaction velocity was quantified by comparing the peak area of the product GlcNAc to a standard curve of GlcNAc at known concentrations. In both cases, the substrate consumption was limited to less than 10%. The *K*_m_ and *k*_cat_ values were also calculated by linear regression of the data using Lineweaver-Burk plots.

TMG-chitotriomycin was kindly provided by Professor Biao Yu (Institute of Organic Chemistry, Chinese Academy of Science). The inhibitory kinetics of TMG-chitotriomycin for the wild-type and mutant forms of OfHex1, SmChb and SpHex were studied using 4MU-β-GlcNAc (4-methylumbelliferone-*N*-acetyl-β-D-glucosaminide, Sigma) as a substrate [8]. The *K*_i_ values were calculated by linear regression of the data using Dixon plots.

### Crystallization and Data Collection

Mutant OfHex1 (E328A) was incubated with excessive TMG-chitotriomycin (5-fold the amount of protein), and then concentrated to ∼7 mg/ml. Vapour diffusion crystallization experiments were set up at 4°C by mixing 1 µl of protein and 1 µl of mother liquor consisting of 100 mM HEPES (pH 7.4), 200 mM MgCl_2_, and 33% PEG400. Diffraction data of mutant OfHex1 (E328A)-TMG-chitotriomycin complex was collected using an in-house Rigaku Micromax-007 HF (Rigaku Raxis IV++ Image Plate, wavelength 1.5418 Å, at 180 K), and processed using the Crystal Clear software package [22].

### Determination and Refinement of Structures

The structure of mutant OfHex1 (E328A)-TMG-chitotriomycin complex was solved by molecular replacement with Molrep [23] using the structure of OfHex1-TMG-chitotriomycin complex (PDB accession number: 3NSN) as the search model. There was one monomer in the asymmetric unit for the structure. Structure refinement was achieved by Refmac5 [24] and CNS [25]. Model building was performed in Coot [26]. The quality of the final model was checked by PROCHECK [27]). All structural figures were prepared by PyMOL.

## Results

### Structure Comparisons between BGlu1-cellotetraose and OfHex1-TMG-Chitotriomycin

Structural comparisons between BGlu1-cellotetraose (PDB accession number: 3F5J) [7] and OfHex1-TMG-chitotriomycin (PDB accession number: 3NSN) [8] were performed to reveal structural differences and similarities.

BGlu1 is a monomeric enzyme with only one catalytic domain while OfHex1 is a dimeric enzyme with an N-terminal zincin-like domain and a C-terminal catalytic domain in each subunit. Although both BGlu1 and OfHex1 possess non-classical catalytic (β/α)_8_-barrels, the overall topology of the two enzymes’ catalytic domain is quite different as supported by the fact that the C^α^ atoms of the catalytic domain of BGlu1 (residues 1 to 476) superimposed on those of OfHex1 (residues 207 to 594) gives a RMSD value of 3.8 Å (based on the overlap of 275 C^α^ atoms) by a Dali pairwise comparison (Fig. 1A) [28]. In addition, BGlu1 has four distinguished loops (Loop A, residues 25–65; Loop B, residues 177–206; Loop C, residues 314–363; Loop D, residues 387–403) while OfHex1, in accordance with BGlu1, has two corresponding loops including *L*_314–335_ (residues 314–335) and *L*_478–496_ (residues 478–496).

By using the pair-fitting alignment using the atoms in the ring of the -1 sugar (5 carbon atoms and 1 oxygen atom) as fitting pairs, the spatial arrangements of the residues comprising the -1 subsites of BGlu1 and OfHex1 are different. Except for the His^130^/Asn^175^/Glu^176^ in BGlu1 and His^303^/Asp^367^/Glu^368^ in OfHex1 constituting the catalytic residue clusters, only two pairs of residues were found to be spatially conserved and with similar functions. Trp^433^ in BGlu1 and Trp^524^ in OfHex1 are located in the bottoms of the -1 subsites and stack with the non-reducing end sugar rings while Gln^29^ in BGlu1 and Glu^526^ in OfHex1 make hydrogen bonds with C4-OH/C6-OH and C4-OH of -1 sugars, respectively (Fig. S1).

However, a striking similarity in the architectures was discovered in the +1 sugar binding sites (Fig. 1B). Pair-fitting alignment was performed by using the atoms in the ring of the +1 sugar (5 carbon atoms and 1 oxygen atom) as fitting pairs. The spatial locations of interactions for binding the +1 sugar are conserved between the two enzymes.

It is interesting to note that the residues involved in the interactions are located in distinguished loops. Tyr^131^, Ile^179^ and Trp^358^ in BGlu1 are located in the loop between the β3 strand and the α3 helix, Loop B (between the β4 strand and the α4 helix) and Loop C (between the β6 strand and the α6 helix), respectively [6], [7]. Accordingly, both Val^327^ and Glu^328^ in OfHex1 are located in *L*_314–335_ while Trp^490^ is located in *L*_478–496_ [8].

Trp^358^, which stacks with the +1 sugar from the down side in BGlu1, corresponds to Trp^490^ in OfHex1. Ile^179^, which forms a hydrophobic interaction with the +1 sugar in BGlu1, corresponds to Val^327^ in OfHex1. Moreover, Tyr^131^, which forms a hydrogen bond with the C3-OH of the +1 sugar via a water molecule in BGlu1, corresponds to Glu^328^ in OfHex1 which directly interacts with the C3-OH of the +1 sugar. The carboxyl oxygen atom of Glu^328^ in OfHex1 is positioned in the same location as a water molecule in BGlu1 which intermediates hydrogen bonding formation between the +1 sugar and BGlu1. One may notice that BGlu1 Glu^440^ also makes a water-mediated hydrogen bond with the C3-OH of the +1 sugar, and is almost close enough to make a direct hydrogen bond (3.4 Å in the 3F5K structure), although it also H-bonds to O4 and O6 of the -1 subsite glycosyl residue. However, OfHex1 does not contain such a residue in the active site. Thus, the spatial conservation of the residues Val^327^/Glu^328^/Trp^490^ in OfHex1 and Ile^179^/Tyr^131^/Trp^358^ in BGlu1 is believed to be functionally important.

### Conservation of Residues in the +1 Subsite among Chitinolytic Enzymes

Structure-based multiple sequence alignments were performed to determine whether the Val^327^, Glu^328^ and Trp^490^ residues in OfHex1 are spatially conserved at the +1 subsite among all chitinolytic β-N-acetyl-D-hexosaminidases. The sequences selected for analysis were taken from representative species including: 1) a choanoflagellate (*Monosiga brevicollis*), a close living relative of animal ancestors [29]; 2) a branchiopod (*Daphnia pulex*), a close relative of the ancestors of higher crustaceans and insects [30]; 3) a crustacean (*Fenneropenaeus chinensis*); 4) an insect (*Drosophila melanogaster*); 5) a fungus (*Candida albicans*) and 6) a plant (*Arabidopsis thaliana*). OfHex1 (PDB accession number: 3NSN) and two bacterial β-N-acetyl-D-hexosaminidases, SmChb from *S. marcescens* (PDB accession number: 1QBB) [16] and SpHex from *S. plicatus* (PDB accession number: 1HP5) [17], were selected as input structures.

**Figure 2:**
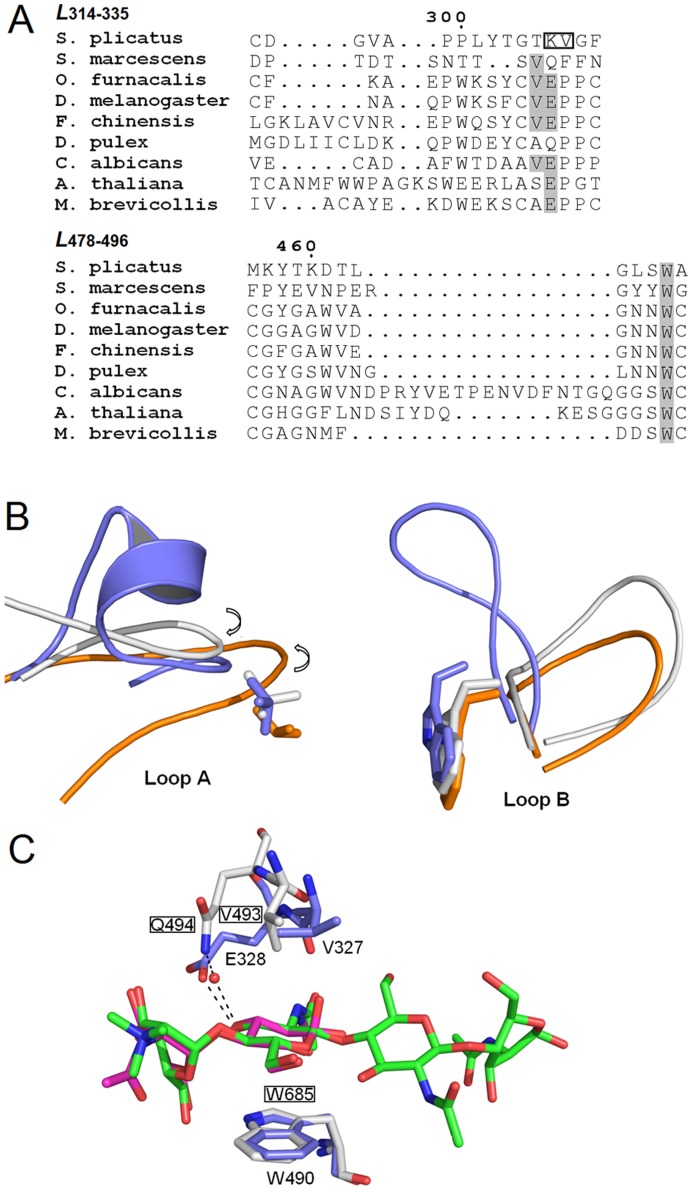
Multiple sequence alignment and structure comparison of chitinolytic β-N-acetyl-D-hexosaminidases. (**A**) Multiple sequence alignments of chitinolytic β-N-acetyl-D-hexosaminidases. They are from *Drosophila melanogaster* (AAF47881), *Fenneropenaeus chinensis* (ABB86961), *Daphnia pulex* (EFX90079), *Candida albicans* (AAA34346), *Arabidopsis thaliana* (NP_172050) and *Monosiga brevicollis* (EDQ91031). The PDB files of OfHex1 (3NSN), SmChb (1QBB) and SpHex (1HP5) were selected as inputted structures. The conserved Val, Glu and Trp residues are shaded. (**B**) Overlapping of Loop A and Loop B segments of three chitinolytic β-N-acetyl-D-hexosaminidases. They are OfHex1 (3NSN, in blue), SmChb (1QBB, in white) and SpHex (1HP5, in orange). The conserved Val and Trp residues are shown as sticks. The directions of loops are shown by arrows. (**C**) Overlapping of the +1 subsites in the active pockets of OfHex1 (in blue) in complex with TMG-chitotriomycin (in green) and SmChb (in white) in complex with (GlcNAc)_2_ (in pink). Water molecules are shown as red balls and hydrogen bonds are shown as black dashes. The residues of SmChb were grounded.

The results of the amino acid sequence alignment indicated that, although the length of the loop varies, Trp^490^ in the loop (*L*_478–496_ in OfHex1) is highly conserved in all of the selected chitinolytic β-N-acetyl-D-hexosaminidases (Fig. 2A, Fig. S2). This demonstrated that the stacking interaction between the +1 sugar and the conserved Trp is vital for the function of the chitinolytic β-N-acetyl-D-hexosaminidases. Compared to the loop *L*_478–496_, the loop *L*_314–335_ varies in both length and amino acid composition, a function of the biodiversity of the species chosen for the sequence alignment (Fig. 2A).

The residues in the *L*_314–335_, namely Val^327^ and Glu^328^ in OfHex1, are also conserved in most β-N-acetyl-D-hexosaminidases (Fig. 2A). Although the enzymes from *S. marcescens* and *D. pulex* possess a Gln instead of Glu^328^, this may be considered a conservative substitution in terms of the similar possibilities for hydrogen bond formation.

Val^327^ was replaced by a smaller residue (Ala or Ser) in the β-N-acetyl-D-hexosaminidases from plant (*A. thaliana*) and the original species (*M. brevicollis* and *D. pulex*), suggesting the species-specific conservation of Val^327^ in bacteria, fungi, shrimps and insects. Moreover, the absence of Val^327^ in enzymes from lower species (*M. brevicollis* and *D. pulex*) implies the existence of a separate evolutionary branch of chitinolytic β-N-acetyl-D-hexosaminidases.

The structural comparison revealed that the direction of the loop (*L*_314–335_ of OfHex1 and SmChb) is clockwise while that of SpHex is counter-clockwise (Fig. 2B). Although the primary structures of *L*_314–335_s and *L*_478–496_s in these enzymes are totally different (Fig. 2A), both Val^327^ and Trp^490^ in OfHex1 can be found in SpHex and SmChb (Val^276^ and Trp^408^ in SpHex, Val^493^ and Trp^685^ in SmChb) (Fig. 2B). For Glu^328^ in OfHex1, SmChb possesses Gln^494^ but SpHex does not possess any comparable residues in this position (Fig. 2C). Glu^328^ in OfHex1 functions through a hydrogen bond with the C3-OH of the +1 sugar. Gln^494^ in SmChb, however, functions the same way as the Tyr^131^ in BGlu1, that is, by interacting with the C3-OH of the +1 sugar via a water-mediated hydrogen-bond (Fig. 1A).

### Significance of the Residues Conserved in the +1 Subsite

To study the role of Val^327^, Glu^328^ and Trp^490^ during substrate binding and catalysis, the enzymatic properties of both the wild-type and mutants of OfHex1 were compared by using β1,4-conjugated (GlcNAc)_2_ and β1,2-conjugated GlcNAcβ1,2Man.

**Figure 3:**
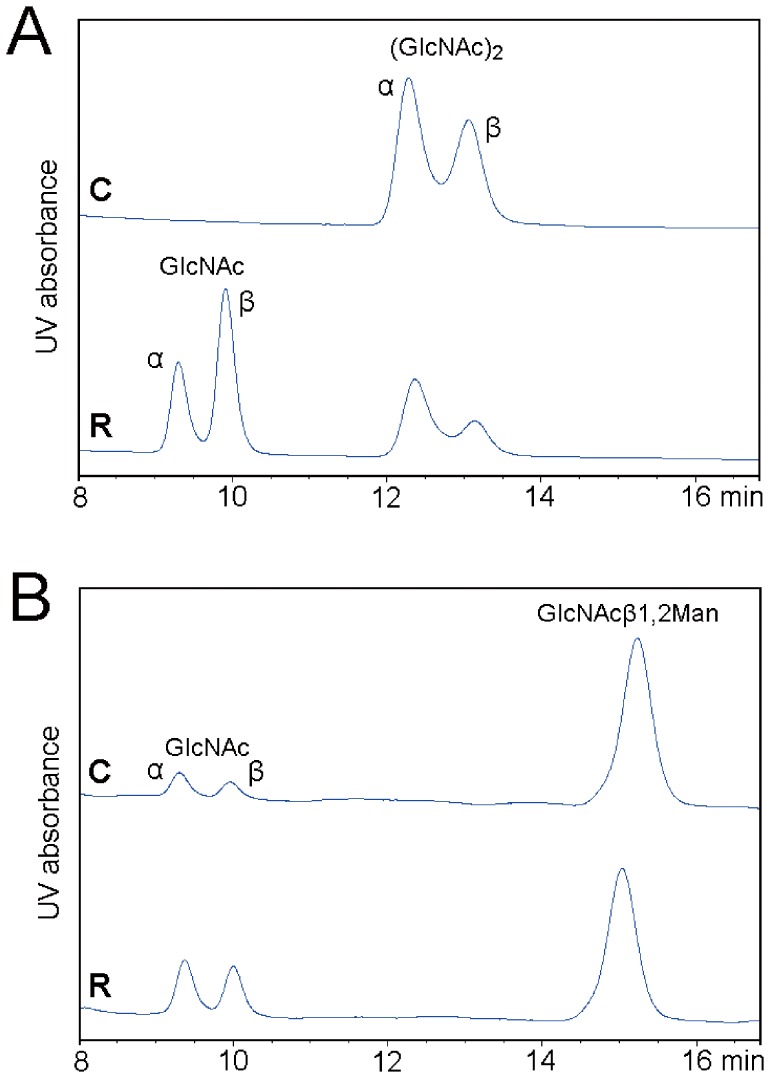
HPLC analysis of the enzymatic hydrolysis of (GlcNAc)_2_ (GlcNAcβ1,4GlcNAc) and GlcNAcβ1,2Man by the V327G mutant of OfHex1. (**A**) Hydrolysis of (GlcNAc)_2_ by the V327G mutant of OfHex1. (**B**) Hydrolysis of GlcNAcβ1,2Man by the V327G mutant of OfHex1. C, control sample; R, reaction sample.

Using (GlcNAc)_2_ as the substrate, the catalytic efficiency after site-directed mutagenesis was determined (Fig. 3A). The *k*_cat_ values of the mutants, V327G, E328Q, E328A and W490A were found to be lower than the wild-type, ranging from 12% (E328Q) to 26% (V327G) (Table 1). The *K*_m_ values of V327G, E328Q, E328A and W490A mutants were 1-, 2.5-, 8.6- and 12.7-fold higher, respectively, than that of the wild-type. Based on the *k*_cat_/*K*_m_ values, the catalytic efficiencies of the mutants for (GlcNAc)_2_ were much lower (from 1.3- to 14.7-fold) than the wild-type. Furthermore, using 4MU-β-GlcNAc as the substrate, the *K*_i_ values of TMG-chitotriomycin for the wild-type and mutants V327G, E328Q, E328A and W490A were determined (Table 2). The *K*_i_ values for TMG-chitotriomycin toward the mutants, V327G, E328Q, E328A and W490A, were increased by 1.1-, 1.6-, 42- and 2277-fold, respectively, compared to the wild-type. It is worthy to note that the mutation of Glu^328^ to Ala instead of Gln seriously impaired the ability of OfHex1 to bind the substrate. These results demonstrated that Glu^328^ and Trp^490^ but not Val^327^ are vital for substrate binding.

**Table 1:** Kinetic parameters of chitinolytic β-N-acetyl-D-hexosaminidases for GlcNAcβ1,4GlcNAc and GlcNAcβ1,2Man.

	(GlcNAc)_2_			GlcNAcβ1,2Man		
	*K*_m_ (mM)	*k*_cat_ (s^-1^)	*k*_cat_/*K*_m_ (s^−1 ^mM^−1^)	*K*_m_ (mM)	*k*_cat_ (s^−1^)	*k*_cat_/*K*_m_ (s^−1 ^mM^−1^)
OfHex1 (WT)	0.15^a^	507^a^	3380^a^	**–** b	**–**	**–**
OfHex1 (V327G)	0.14	375	2536	0.54	0.38	0.70
OfHex1 (E328Q)	0.38	446	1174	**–**	**–**	**–**
OfHex1 (E328A)	1.29	404	313	**–**	**–**	**–**
OfHex1 (W490A)	1.90	437	230			
SmChb	0.50	284	568	–	–	–
SpHex	2.70	1330	493	–	–	–

a Data from [8].

b Not detected.

**Table 2:** *K*_i_ values of TMG-chitotriomycin for GH20 β-N-acetyl-D-hexosaminidases.

	*K*_i_ (µM)
OfHex1 (WT)	0.065^a^
OfHex1 (V327G)	0.077^a^
OfHex1 (E328Q)	0.101
OfHex1 (E328A)	2.714
OfHex1 (W490A)	148^a^
SmChb	0.077^b^
SpHex	1^b^

a Data from [8].

b Data from [10].

To better understand the function of Val^327^, additional substrates were studied. As OfHex1 has evolved to degrade β1,4-glycosidic bonds in linear chitooligosaccharides, it is surprising to observe that the mutation of Val^327^ to Gly results in OfHex1 having the remarkable capability to hydrolyze the substrate GlcNAcβ1,2Man (Table 1 and Fig. 3B). The catalytic efficiency of V327G will be further explored in the discussion section.

### Crystal Structure Analysis of E328A Complexed with TMG-chitotriomycin

**Figure 4:**
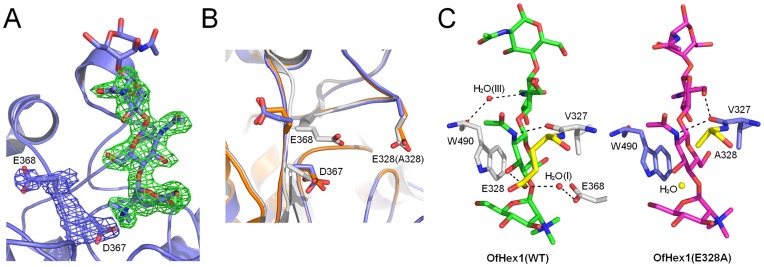
Structure comparison of wild-type OfHex1 and mutant OfHex1 (E328A). (**A**) The structures of mutant OfHex1 (E328A) in complex with TMG-chitotriomycin. The unbiased |*F*_O_| - |*F*_C_|, *φ*_calc_ electron density map contoured at 3.0 σ, used for building the models of TMG-chitotriomycin, is shown in green and the unbiased 2|*F*_O_| - |*F*_C_|, *φ*_calc_ electron density maps contoured at 1.0 σ, used for building the models of Asp^367^ and Glu^368^, are shown in blue. (**B**) Overlapping of the residues with conformational changes of wild-type OfHex1 (*apo-*structure, in yellow; complex structure, in white) and mutant OfHex1 (E328A) (in blue). (**C**) Intermolecular interactions between wild-type OfHex1 and mutant OfHex1 with TMG-chitotriomycin. Water molecules are shown as balls and hydrogen bonds are shown as black dashes.

To reveal the structural basis behind the biochemical data, crystal structural information is needed. Since we have obtained the structure of OfHex1 V327G mutant previously [12], in this study, the crystallization of E328A in complex with TMG-chitotriomycin was performed and the complex structure was resolved to 2.5 Å. The statistics of data collection and structure refinement are summarized in Table 3. The coordinates were deposited in the Protein Data Bank with accession number 3VTR. The structure showed well defined unbiased map for TMG-chitotriomycin (except GlcNAcIII at the reducing end) and side chains of Asp^367^ and Glu^368^ (Fig. 4A).

**Table 3:** Details of data collection and structure refinement.

	OfHex1(E328A)-TMG-chitotriomycin
**Data collection**	
Space group	*P* 3_2_21
Cell dimensions	
*a*, *b*, *c* (Å)	107.9, 107.9, 174.9
*α*, *β*, *γ* (°)	90.0, 90.0, 120.0
Resolution (Å)	53.97-2.50 (2.59-2.50)^a^
*R*_sym_ or *R*_merge_	0.110 (0.376)
*I/σI*	6.6 (2.2)
Completeness (%)	99.3 (98.7)
Redundancy	4.22(4.27)
**Refinement**	
Resolution (Å)	2.50
No. reflections (total)	173,820
No. reflections (unique)	41,167
*R*_work_/*R*_free_	0.214/0.244
No. of atoms	
Protein	4611
Ligand/ion	85
Water	254
*B*-factors	
protein	23.0
ligand	33.3
water	24.4
Root mean square deviations	
Bond lengths (Å)	0.009
Bond angles (°)	1.137
Ramachandran plot	
Most favoured (%)	91
Additionally allowed (%)	9
**PDB code**	3VTR

a Values in parentheses are for the highest resolution shell.

Structure alignment of the OfHex1-TMG-chitotriomycin complex with the E328A-TMG-chitotriomycin complex was performed using PyMOL. According to the alignment, the mutation of Glu^328^ to Ala resulted in obvious conformation changes (Fig. 4B). The catalytic residues, Glu^368^ and Asp^367^ are rotated about 180° and 90° compared to those in the OfHex1-TMG-chitotriomycin complex but with similar conformations as those in the *apo*-structure of OfHex1. The rotation of Glu^368^ led to the collapse of the two important hydrogen bond networks (Asp^249^-His^303^-Glu^368^ and Glu^328^-H_2_O (I)-Glu^368^) [8]. In addition, the mutation of Glu^328^ to Ala increased the volume of the active pocket from 646.8 Å^3^ to 758.3 Å^3^ as measured with CASTp [31], suggesting an important role of Glu^328^ in constructing the active pocket.

The conformation of TMG-chitotriomycin, in particular the GlcNAcII moiety, was found to be very different from that observed in the wild-type (Fig. 4C). Although the sugar rings of GlcNAcII in both complexes are in the ^1^ *C*_4_ conformation and are superimposable, the C5- hydroxymethyl groups of GlcNAcII orient to different directions.

In addition, the conformation of GlcNAcIII in OfHex1 is ^O^ *S*_2_, but the electron density was not clear enough to identify its conformation in OfHex1 (E328A). However, there is no obvious minus peaks found around GlcNAcIII with the current conformation (^3,O^ *B*) in the difference map. Therefore, this unclearness of the electron density map can be considered as the result of the flexibility of GlcNAcIII.

Furthermore, mutation of Glu^328^ to Ala leads to changes in polar interactions between the wild type and the mutant when complexed with TMG-chitotriomycin (Fig. 4C). The most obvious change is the absence of short polar interaction between Glu^328^ and the C3-OH of GlcNAcI of TMG-chitotriomycin. Interestingly, a water molecule was found to occupy the position of the side chain of Glu^328^.

Several other changes in intermolecular polar interactions were also observed in the E328A-TMG-chitotriomycin complex when compared to the wild type (Fig. 4C). Firstly, the distance between the nitrogen atom of the 2-acetamido group (GlcNAcI) and the oxygen atom of the carbonyl group (main chain of Val^327^) increased from 2.8 Å to 3.2 Å. The interaction between the nitrogen atom of the 2-acetamido group (GlcNAcII) and the oxygen atom of the carbonyl group (main chain of Trp^490^) is missing, but instead, C6-OH of GlcNAcII forms a hydrogen bond with the main chain of Val^327^.

## Discussion

Enzymes capable of degrading cellulose and chitin are undoubtedly significant because these saccharides are the two most abundant forms of biomass in nature. Since cellulose and chitin are similar in both their chemical structure and catabolic procedures, it can be deduced that the associated hydrolases would share some commonalities, for example, their substrate binding mechanism or glycosidic bond preference. A structural comparison may provide new clues to uncover the evolutionary divergence of these enzymes between plants and animals as well as provide supporting information for the use of their associated biomass in industrial or medical applications. However, to date, little work has been performed with regard to comparing cellulolytic enzymes with chitinolytic enzymes. Herein, we investigated the structures of a plant cellulolytic enzyme (BGlu1) and an insect chitinolytic enzyme (OfHex1) in an attempt to find their similarities and differences.

A structural comparison revealed that both GH1 BGlu1 and GH20 OfHex1 possess three residues essential for binding the +1 sugar of substrates [6]–[8]. Site-directed mutagenesis, enzyme kinetics as well as crystal structure determination of OfHex1 revealed that Glu^328^ and Val^327^ are both essential residues for determining glycosidic bond specificity and the +1 sugar binding, respectively.

### BGlu1Tyr^131^ vs. OfHex1Glu^328^

Both Tyr^131^ (BGlu1) and Glu^328^ (OfHex1) residues form hydrogen bonds with the C3-OH of the +1 sugar. Tyr^131^ (BGlu1), the residue conserved in most plant cellulolytic β-glucosidases [6], [7], interacts with the +1 sugar in BGlu1 via a water molecule. Accordingly, OfHex1 Glu^328^, the residue conserved or conservatively replaced (e.g., by Gln) in chitinolytic β-N-acetyl-D-hexosaminidases (Fig. 2A), directly interacts with the +1 sugar in OfHex1. Like BGlu1 Tyr^131^, the conservatively replaced residue, Gln^494^, in SmChb also interacts with the +1 sugar via a water molecule.

Site-directed mutagenesis indicated the change of Glu^328^ to Ala impaired OfHex1’s affinity toward the substrate, (GlcNAc)_2_, by 8-fold and the inhibitory activity of TMG-chitotriomycin by 42-fold (Tables 1 & 2). However, the mutation of Glu^328^ to Gln only caused a slight impairment in binding (Tables 1 & 2). Thus, we deduced that the impairment is most likely caused by the absence of a strong polar interaction (2.67 Å) between Glu^328^ and GlcNAcI (Fig. 4C).

Moreover, using (GlcNAc)_2_ as the substrate, the *K*_m_ values of SmChb (with Gln^494^ corresponding to Glu^328^ of OfHex1) is in accordance with that of E328Q, and the *K*_m_ values of SpHex (no residue at the corresponding spatial location) is in accordance with that of E328A (Table 1). These results further confirmed the involvement of Glu^328^ in substrate binding of OfHex1.

Taken all together, since all the chitinolytic enzymes including SmChb, SpHex and OfHex1 contain the conserved Val and Trp residues at their +1 sugar binding sites, the difference in their affinities for (GlcNAc)_2_ is most likely because of the presence of Glu^328^ (in OfHex1). The appearance of a conserved Glu at the +1 sugar binding sites might be the result of positive evolution.

### BGlu1 Ile^179^ vs. OfHex1 Val^327^

Both Ile^179^ (BGlu1) and Val^327^ (OfHex1) function at the +1 subsites by sandwiching the +1 sugar together with the residue Trp^358^ (BGlu1) and Trp^490^ (OfHex1), respectively. Sequence alignment indicated that the BGlu1 Ile^179^ is replaced by Val in the plant cellulolytic β-glucosidase BGQ60 from *Hordeum vulgare* [32]–[35]. Accordingly, OfHex1 Val^327^ was conserved in most chitinolytic β-N-acetyl-D-hexosaminidases, although there are several exceptions from *A. thaliana*, *M. brevicollis* and *D. pulex*, the corresponding residue of which is replaced by Ser or Ala.

As indicated by the *K*_m_ values for cellobiose, BGQ60 has much higher binding affinity at +1 subsite than BGlu1 [32]–[35]. But the mutation of Ile^179^ to Val does not improve BGlu1’s affinity for cellobiose, indicating Ile^179^ is not essential for the cellobiose binding [6]. Accordingly, mutation of Val^327^ to Gly did not impair OfHex1’s affinity for the substrate [(GlcNAc)_2_] and the inhibitor (TMG-chitotriomycin) (Tables 1 & 2). These results lead us to believe that OfHex1 Val^327^ may not be so crucial for substrate binding (Fig. 2A).

However, a dramatic conformational change in Val^327^ is observed after OfHex1 binds TMG-chitotriomycin [8], namely the isopropyl group of Val^327^ moves from a vertical position to a parallel position around the indolyl plane of Trp^490^. As a result, the entrance size of the active pocket is enlarged from 7.25 Å to 8.26 Å. Thus, we assumed that this conformation change may suggest that Val^327^ plays some other essential role.

Based on the *k*_cat_/*K*_m_ values, although V327G hydrolyses GlcNAcβ1,2Man 3623-fold slower than (GlcNAc)_2_, it is still 9% faster than StrH. StrH is a bacterial β-N-acetyl-D-hexosaminidase from *Streptococcus pneumonia* that is believed to be able to specifically hydrolyse β1,2-linked GlcNAc substrates [36]. According to the structural comparison of OfHex1 wild-type, V327G and StrH (GH20A-GlcNAcβ1,2Man complex), OfHex1 has a narrow active site pocket (Fig. S3A) which constrains the +1 GlcNAc sugar via hydrophobic and polar interactions with Val^327^, Glu^328^ and Trp^490^ (Fig. 4C). However, StrH (GH20A) has a shallow active pocket (Fig. S3A) in which the +1 Man is almost solvent exposed [37]. Furthermore, as shown in Fig. S3B, the conformations of both the +1 mannose in the StrH complex and the +1 GlcNAc in the OfHex1 complex are ^1^ *C*_4_ chairs, but the two superimposed sugar ring is separated by a 35.4° dihedral angle [37]. Thus, if GlcNAcβ1,2Man binds to the active pocket of OfHex1, steric hindrance might be encountered between the C3-OH and C6-OH of the +1 Man and the isopropyl group of Val^327^. This steric hindrance is removed by the mutation of Val^327^ to Gly. Thus, V327G is conferred with the ability to catalyse the hydrolysis of GlcNAcβ1,2Man. On the basis of the above findings, Val^327^ was speculated to have a role in the β1,4 glycosidic bond specificity of chitinolytic β-N-acetyl-D-hexosaminidases.

In summary, two conserved residues in chitinolytic β-N-acetyl-D-hexosaminidases with totally different functions were discovered by comparative structural analysis between the insect chitinolytic β-N-acetyl-D-hexosaminidase OfHex1 and the plant cellulolytic β-glucosidase BGlu1. OfHex1Glu^328^ is vital for the binding of chitooligosaccharides while OfHex1Val^327^ is responsible for the glycosidic bond specificity. Together with the previously determined role of Trp^490^ in binding chitooligosaccharides, the functions of all the three conserved residues comprising the +1 subsite of chitinolytic β-N-acetyl-D-hexosaminidases have been revealed.

## Supporting Information

Figure S1 **Structural comparison of residues comprising the -1 subsites of BGlu1 (A) and OfHex1 (B).** The residues with similar spatial locations and functions are underlined.
(DOC)

Figure S2 **Sequence alignment of chitinolytic GH20 β-N-acetyl-D-hexosaminidases.**
(DOC)

Figure S3 **Structure comparison of wild-type OfHex1, mutant OfHex1 (V327G) and StrH.** (**A**) Comparison of the active-pocket architectures of OfHex1 and StrH. TMG-chitotriomycin and GlcNAcβ1,2Man are shown in green and magenta, respectively. (**B**) Comparison of OfHex1 (in white) in complex with TMG-chitotriomycin and V327G (in blue) in complex with GlcNAcβ1,2Man (A model obtained by superposition of the -1 sugars of GlcNAcβ1,2Man in StrH complex and TMG-chitotriomycin in OfHex1 complex and superposition of wild-type OfHex1 and V327G).
(DOC)
