# Supplementary figures and images for: Structural Insights into Cellulolytic and Chitinolytic Enzymes Revealing Crucial Residues of Insect β-N-acetyl-D-hexosaminidase
Source: PLoS One. 2012 Dec 27;7(12):e52225. doi: 10.1371/journal.pone.0052225 (PMC3531433; doi:10.1371/journal.pone.0052225)

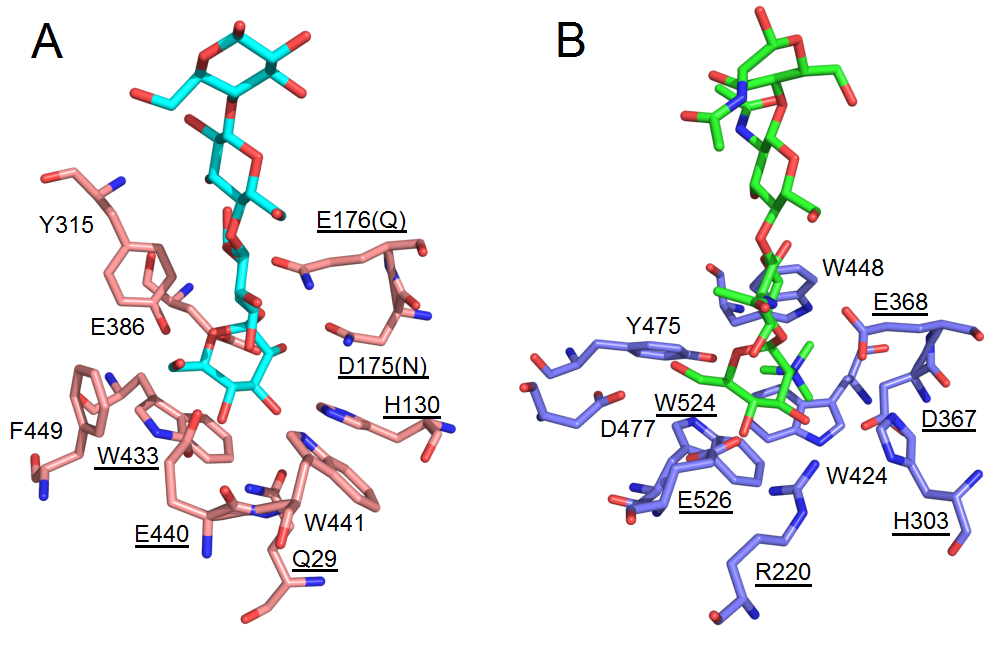

Supplement: Figure S1 — Structural comparison of residues comprising the -1 subsites of BGlu1 (A) and OfHex1 (B). The residues with similar spatial locations and functions are underlined. (DOC) [file pone.0052225.s001.doc]

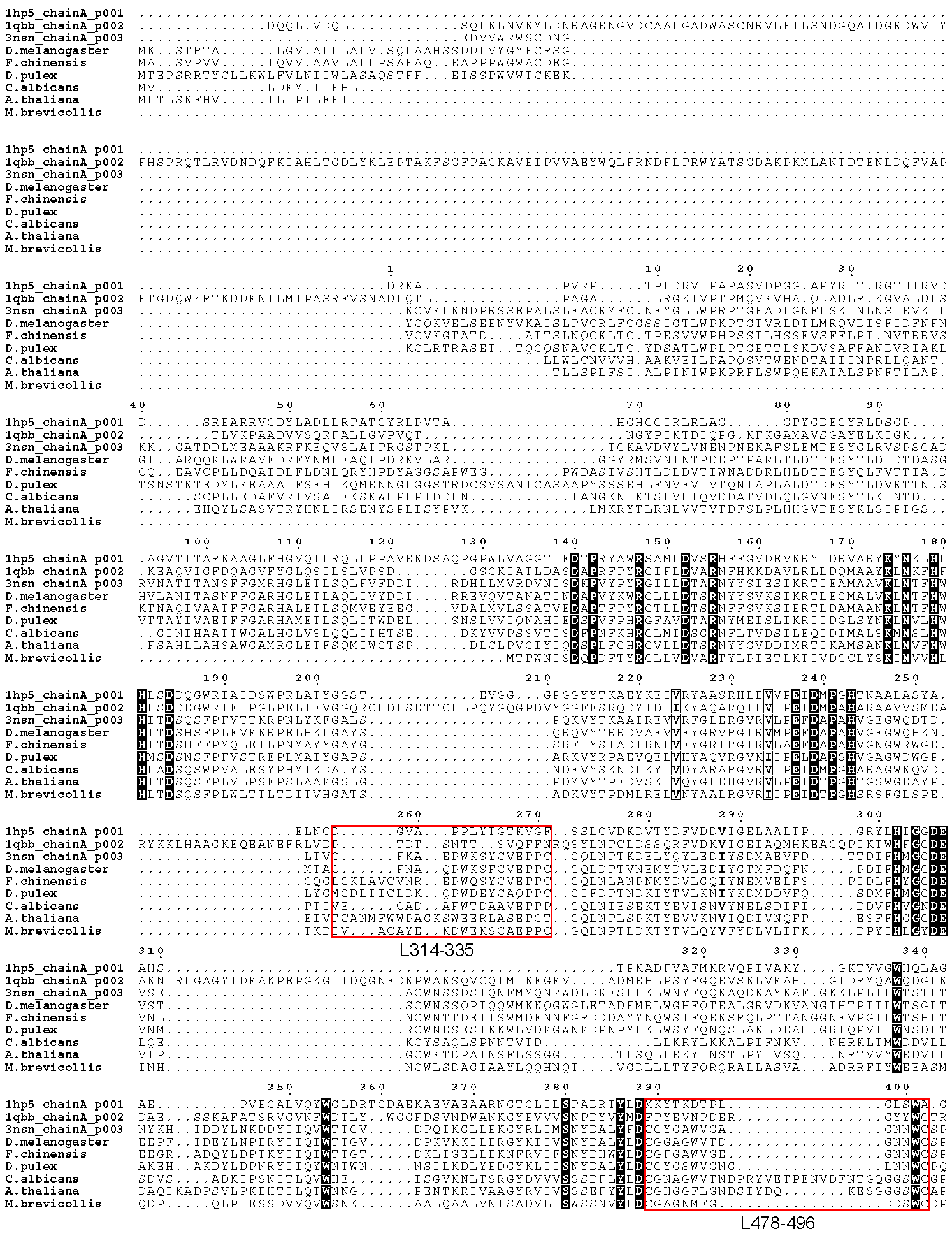


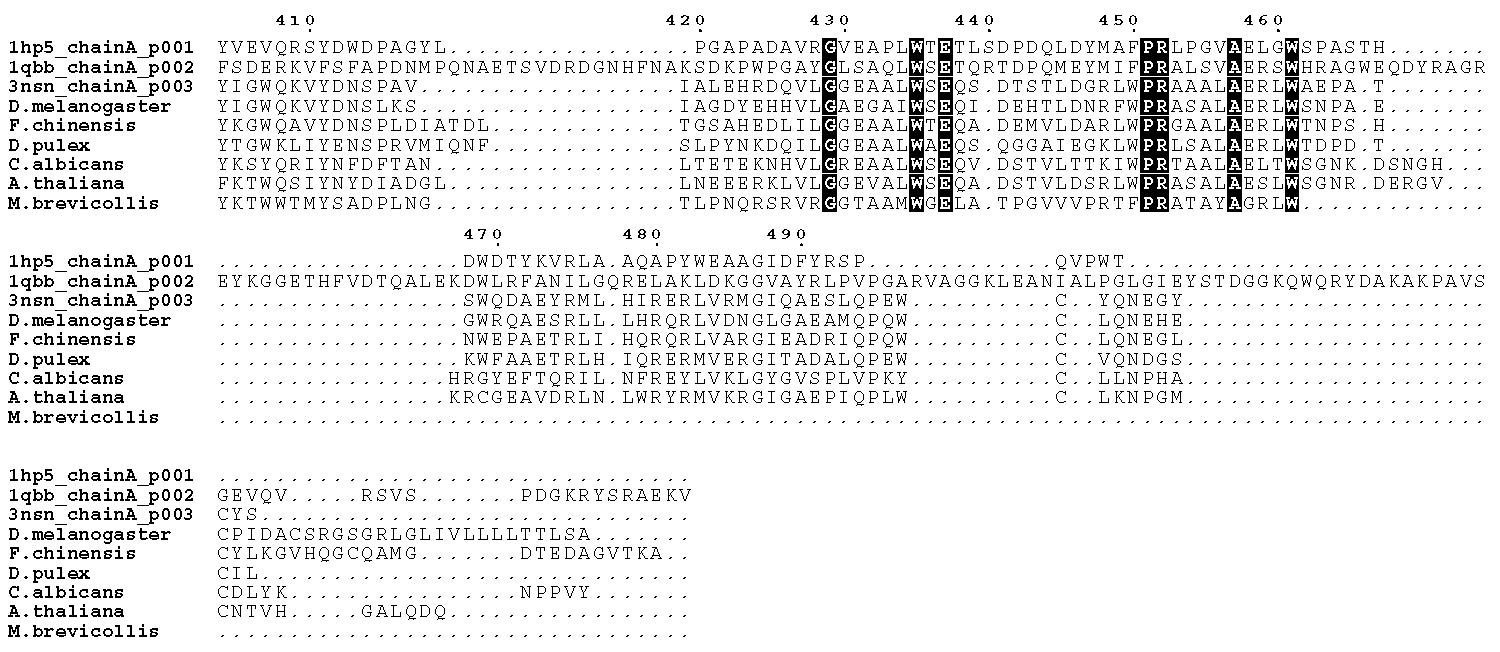

Supplement: Figure S2 — Sequence alignment of chitinolytic GH20 β-N-acetyl-D-hexosaminidases. (DOC) [file pone.0052225.s002.doc]

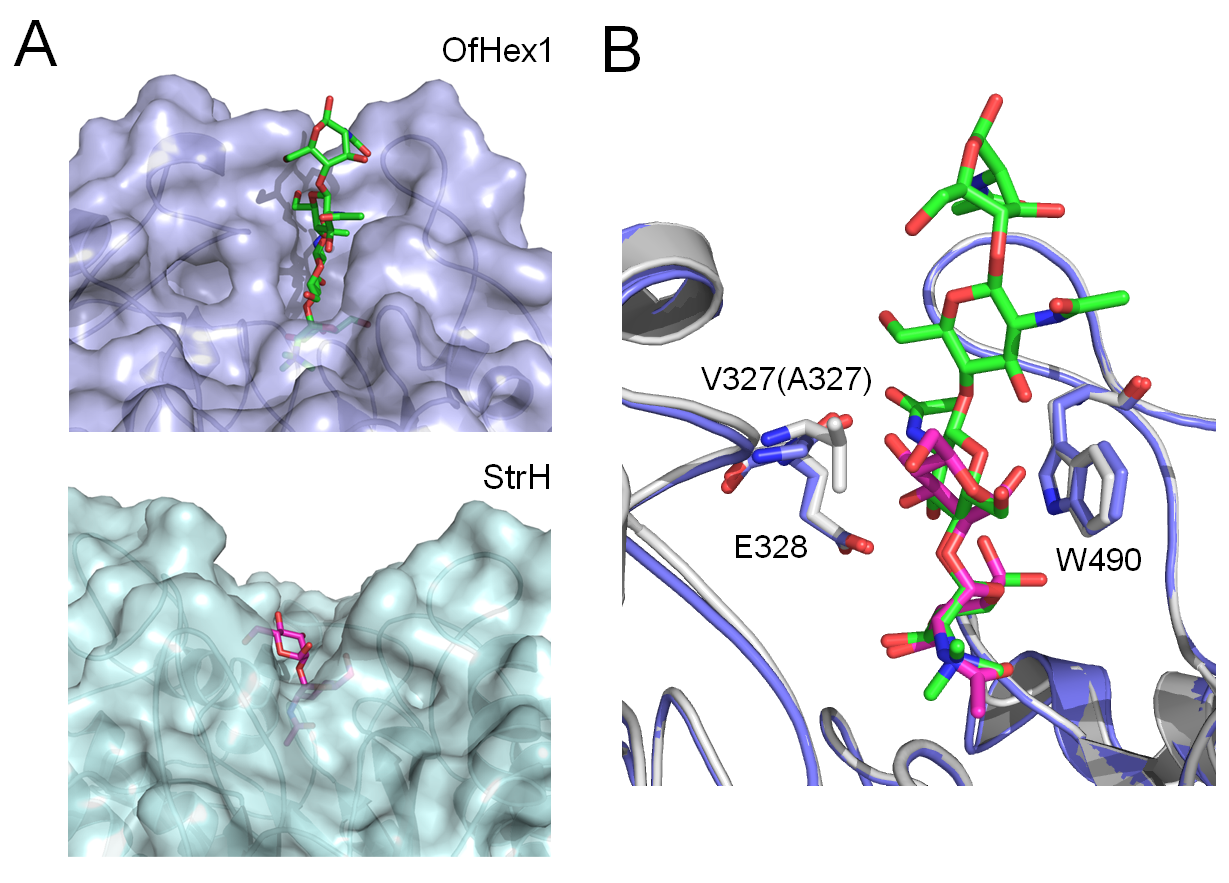

Supplement: Figure S3 — Structure comparison of wild-type OfHex1, mutant OfHex1 (V327G) and StrH. (A) Comparison of the active-pocket architectures of OfHex1 and StrH. TMG-chitotriomycin and GlcNAcβ1,2Man are shown in green and magenta, respectively. (B) Comparison of OfHex1 (in white) in complex with TMG-chitotriomycin and V327G (in blue) in complex with GlcNAcβ1,2Man (A model obtained by superposition of the -1 sugars of GlcNAcβ1,2Man in StrH complex and TMG-chitotriomycin in OfHex1 complex and superposition of wild-type OfHex1 and V327G). (DOC) [file pone.0052225.s003.doc]
